# Do phase-dependent life history traits in cyclic voles persist in a common environment?

**DOI:** 10.1007/s00442-019-04410-3

**Published:** 2019-05-07

**Authors:** Janne Sundell, Hannu Ylönen, Marko Haapakoski

**Affiliations:** 1 Lammi Biological Station, University of Helsinki, Pääjärventie 320, 16900 Lammi, Finland; 2 Department of Biological and Environmental Science and Konnevesi Research Station, University of Jyväskylä, P.O. Box 35, 40014 Jyväskylä, Finland

**Keywords:** Bank vole, Body size, Chitty effect, Maternal effect, Phenotypic plasticity, Common garden

## Abstract

Phenotype and life history traits of an individual are a product of environmental conditions and the genome. Environment can be current or past, which complicates the distinction between environmental and heritable effects on the phenotype in wild animals. We studied genome–environment interactions on phenotype and life history traits by transplanting bank voles (*Myodes glareolus*) from northern and southern populations, originating from low or high population cycle phases, to common garden conditions in large outdoor enclosures. The first experiment focused on the persistence of body traits in autumn-captured overwintering populations. The second experiment focused on population growth and body traits in spring-captured founder voles and F1 generation. This experiment lasted the breeding season and subsequent winter. We verified phase-dependent differences in body size at capture. In the common environment, adult voles kept their original body size differences both over winter and during the breeding season. In addition, the first generation born in the common environment kept the size distribution of their parent population. The increase phase population maintained a more rapid growth potential, while populations from the decline phase of the cycle grew slower. After winter, the F1 generation of the increasing northern population matured later than the F1 of the southern declining ones. Our results suggest a strong role of heredity or early life conditions, greater than that of current juvenile and adult environmental conditions. Environmental conditions experienced by the parents in their early life can have inter-generational effects that manifest in offspring performance.

**Electronic supplementary material:**

The online version of this article (10.1007/s00442-019-04410-3) contains supplementary material, which is available to authorized users.

## Introduction

An individual’s phenotype is the result of both genes and environment. However, it is often difficult to evaluate the relative roles of these in wild populations. One way to get a partial answer is to relocate individuals and observe whether the traits evolved in the original environment are changing in a new environment. If traits exhibit change, environmental conditions are playing a large role in the modification of individual phenotypes. This kind of phenotypic plasticity is commonly observed in many organisms (Schlichting 1986; DeWitt and Scheiner 2004; Whitman and Ananthakrishnan 2009). On the other hand, if traits are strongly determined by a genetic component, no changes in traits are expected, even if there is a substantial change in environmental conditions. A similar outcome can result from prenatal and/or postnatal environment conditions experienced during early development (Lindström 1999; Burton and Metcalfe 2014) which predetermines the later phenotype and life history traits of an individual (e.g., Monaghan 2008). The importance of the roles of genes and environment is trait specific, so that in some traits the inherited component is more important than in others (Taborsky 2006; Helle et al. 2012; Oksanen et al. 2012).

Short-lived northern voles face very different environmental conditions depending on when they have been born. These iteroparous animals experience different environments depending on the time point they have been born within a breeding season, but especially during which phase of the regular multiannual population cycle they are born (Hansson and Henttonen 1988; Sundell et al. 2004). During the density cycle, individual voles experience very different biotic environmental conditions. Density itself potentially plays a large role as the intraspecific competition and conspecific interactions vary greatly with density. Furthermore, since there is synchrony between populations of different small rodent species (e.g., Korpimäki et al. 2005), interspecific competition varies accordingly. The differing high grazing pressure on plants in some years may also affect the amount and/or quality of food available (Huitu et al. 2003). Voles are the most important food resource for many predators and, therefore, predator numbers track the numbers of their vole prey (Sundell et al. 2004; Hellstedt et al. 2006). This is manifested in the cyclic change in predation pressure (predator:prey ratio), which should be highest in the decline phase of the vole cycle (e.g., Hanski et al. 2001; Sundell et al. 2013).

Many physiological or even behavioural traits of voles are suggested to be changing along the density cycle (Krebs and Myers 1974). Perhaps the most striking trait is the body size of voles. The phenomenon known as “Chitty effect” describes changes in vole size; voles during the peak and increase phases are larger than voles from decline/low phases (Chitty 1952, 1967; Boonstra and Krebs 1979; Sundell and Norrdahl 2002). Both the generality and causality of this phenomenon are still controversial. Debate is still going on asking whether body size difference is caused by the intrinsic properties of individuals as Chitty (1952) originally proposed or by extrinsic ones, or perhaps an interaction of these. Extrinsic properties can be divided into those selecting for survival of different body sizes (Hansson and Jaarola 1989; Yoccoz and Mesnager 1998; Sundell and Norrdahl 2002), different allocation of resources between somatic growth or reproduction and survival (Yoccoz and Mesnager 1998; Oli 1999; Johannesen and Andreassen 2008), or environmental conditions limiting the body size (Lidicker and Ostfeld 1991).

Here, we report the results from a study focusing on the relative roles of environment and genes in determining fitness related traits using a common boreal rodent, the bank vole (*Myodes glareolus*), as a model species. We conducted two experiments focusing on body size, breeding and survival traits of voles from different geographical origins, representing different phases of the vole cycle. The first experiment focused on winter survival and body traits of voles that were captured in autumn and brought to overwinter under common garden conditions in large outdoor enclosures in Konnevesi, Central Finland. Voles for the second experiment were captured in spring at the same original sites as the first ones. This experiment focused on the persistence of body size and reproductive differences in the breeding population over the summer. Furthermore, the populations of the founder voles and their offspring were monitored over the next winter.

We hypothesized that if vole traits are mainly determined by current environmental factors, voles from different origin and cycle phases should resemble each other soon after release into a common environment (Ergon et al. 2001a). If voles maintain their original traits in a new common environment, either these are determined mainly by heredity, or by natal environment or maternal effects (i.e., the environment parents have experienced). Furthermore, if the original dissimilarity in traits of the parent population remains in the common environment, but their offspring (F1 generation) become similar we can rule out genetic effects.

## Methods

### Origin of experimental animals

**Fig. 1:**
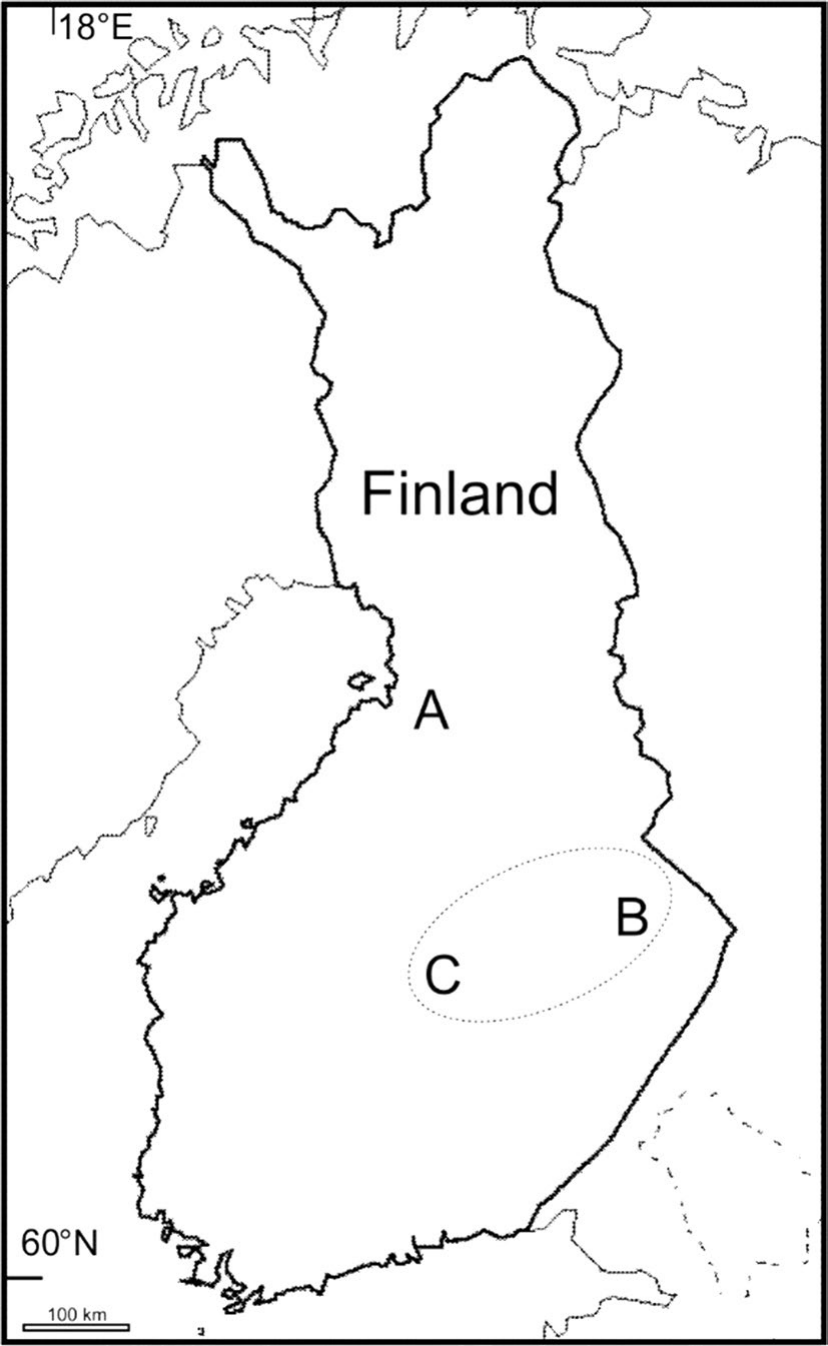
The map shows the areas of original populations: A: Muhos is called North, and B: Koli and C: Konnevesi (enclosed in the ellipse) are combined to South in the analyses of experiments

**Fig. 2:**
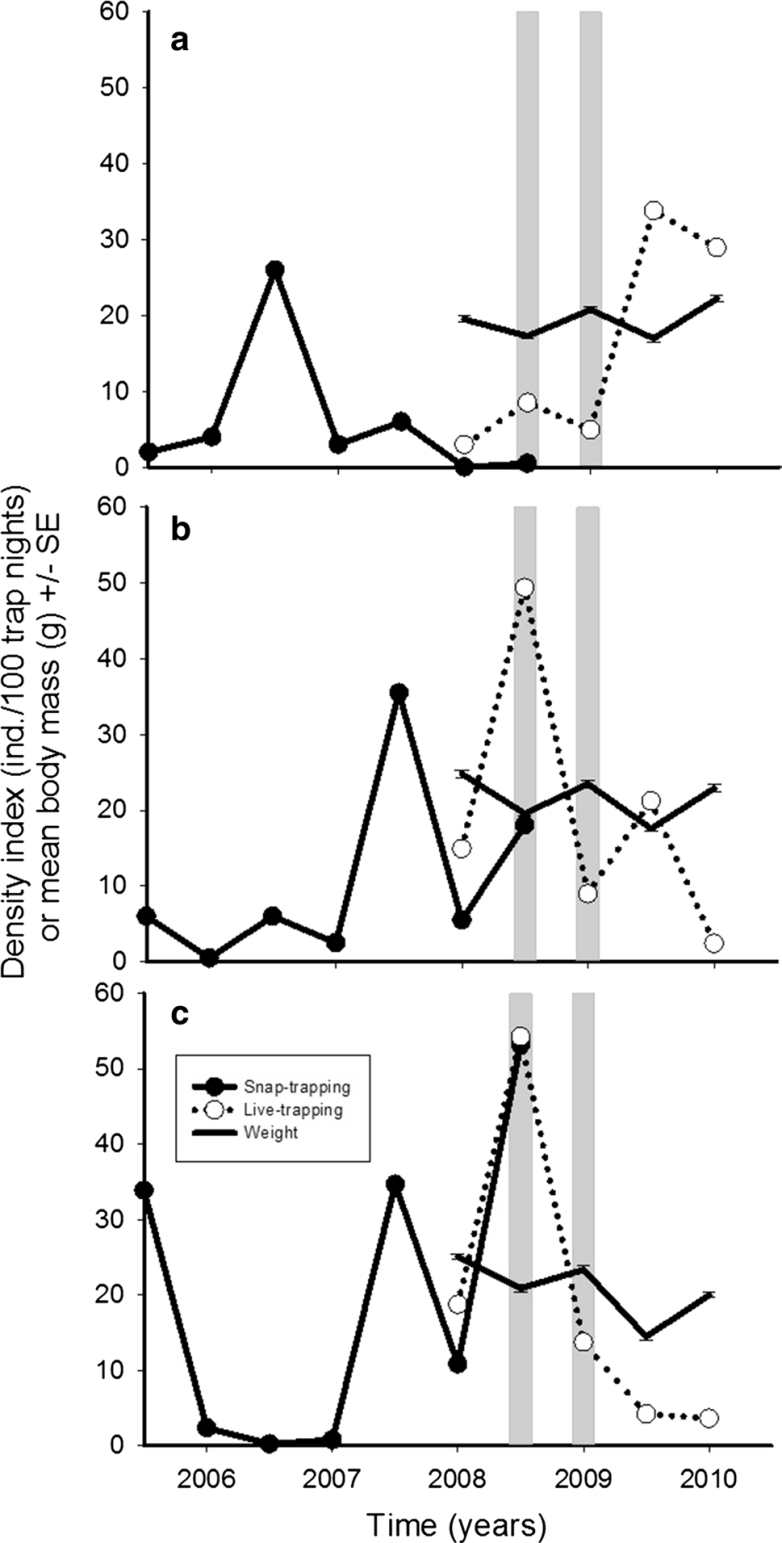
Population dynamics of original populations. **a**–**c** Different areas shown in map of the Fig. 1. Solid lines with dots are from independent biannual (spring and autumn) vole population monitoring of the Finnish Forest Research Institute (snap-trapping) and dotted lines are based on trappings of this study (live-trapping). Vole abundance is expressed as a density index (individuals trapped per 100 trap nights). Solid lines with error bars (± SE) show the development of mean weights of male voles in original populations. Voles for the experiments were captured with live-traps in autumn 2008 and spring 2009. These time points are shown by gray vertical bars

Our aim was to capture voles from different phases of the vole cycle and relocate them into a new common environment. Based on the vole monitoring program of the Finnish Forest Research Institute, we chose three geographically distinct populations for sampling. The sites were Muhos (64°48′N, 25°59′E), Koli (63°07′N, 29°46′E) and Konnevesi (62°37′N, 26° 17′E. The distance between these areas varies from 190 to 260 km (Fig. 1). The history of population dynamics suggested that all these areas were in different phases of the cycle, but our own trappings revealed that, in fact, the Koli and Konnevesi populations were in the same phase, which was different from the northern Muhos population. Samplings of the study populations for the first experiment were conducted in September 2008 and for the second experiment in May 2009. In September 2008, vole populations were in the low phase in Muhos and in the peak phase in the Koli and Konnevesi study areas, while the situation was advanced in May 2009, so that the Muhos population was in the early increase phase and the Koli and Konnevesi populations were in the decline phase of the cycle (Fig. 2).

We chose the most common vole species, the bank vole, which formed 86% of the total number of captured animals, as our study species. Voles were captured with Ugglan special^®^ live-traps (Grahnab, Hillerstorp, Sweden) that were baited with sunflower seeds. Voles were captured from their core habitat, mature forests and edge habitats between forest and field. Voles were weighed at capture to an accuracy of 0.1 g. In addition, head width was measured using a digital caliber with an accuracy of 0.01 mm. To increase repeatability, all head measurements were done by same person (MH). Voles’ maturity status was checked by visual inspection of the genital area. Females with open vagina and males with scrotal testes were categorized as mature. Visibly pregnant females and those which later gave birth in the laboratory were excluded from all the analyses on body mass.

Prior to the experiments, voles were housed singly in standard mouse cages (43 × 26 × 15 cm^3^) with wood shavings and dry hay as bedding material in the laboratory of the Konnevesi Research Station. Water and rodent pellets were provided ad lib.

### Overwintering experiment in a common environment: body size and overwintering success of parental populations

Approximately 1 month after being captured in September 2008, voles were released into 12 outdoor enclosures of 0.25 ha each. Enclosures were built in an old abandoned field situated in Konnevesi, in the same region as one of the study populations. The habitat in all enclosures was generally similar with respect to vegetation and terrain. Vegetation was mainly thick tall grass of family Poaceae (such genera as *Phleum*, *Festuca*, and *Deschampsia*) with some *Chamerion*, *Anthriscus sylvestris*, *Urtica dioica* and *Salix* spp. bushes. The height of vegetation was approximately 0.5–1.0-m during summer. Although the bank vole is a forest-dwelling species, it is also commonly found in abandoned fields and other grassy habitats, especially if its dominant competitor, the field vole (*Microtus agrestis*) is absent or rare (Hansson 1983; Sundell et al. 2012). At the peak, bank voles densities are typically 50–100 individuals/ha in forest habitats (Johnsen et al. 2017). Fences of the enclosures were made of metal sheet that extended about 0.5 m below and 1 m above the ground. Fences effectively restricted movements of voles between enclosures and prevented the entry of small mammalian predators. No tracks of larger predators were observed during the snowy period. The entry of avian predators was not restricted and they were observed especially during the spring and autumn migration. Twelve experimental populations in 12 enclosures were formed so that there were four populations from each study area. Four females and four males were released per enclosure. Voles for enclosures were chosen so that the average weights for both sexes in each enclosure within a study area were approximately the same. For example, the average body mass of males from North was approximately the same in all four enclosures. The age distribution of this autumn population was unknown but none of the individuals were juvenile and all of them were > 14.5 g. The enclosures for each experimental population were chosen randomly.

Voles were monitored by trappings in December 2008, and February, March and April 2009 to evaluate survival, population development and breeding. Sixteen to 25 traps (Ugglan special^®^ live-traps) were placed evenly within each of the enclosures. Traps, baited with oats and sunflower seeds, were checked 8 h intervals until no new or unmarked (with ear tags) animals entered traps anymore. Trappability of bank voles is good and all animals were captured at low density with three trap checks and at high density with five checks. During the last trapping session in the end of April, enclosures were emptied with intensified trappings and all animals were transferred to the laboratory for measurements of weight and head width blindly without knowing their origin.

### Breeding and overwintering in the common environment: body size, population dynamics and maturation of F1 generation

The second experiment was conducted in the same enclosures as the first one from May 2009 to April 2010. The setup was similar except that voles for this experiment were captured in May and were transferred to the enclosures in June. All experimental voles were over-wintered and mature. Voles were monitored by trappings twice in July, once in August and September 2009 and finally in early April 2010, when voles were transferred to the laboratory for measurement of weight. Heads were not measured. Monitoring included parental populations and their F1 generation offspring.

### Statistical analyses

All statistical analyses were conducted with R version 3.3.0 (R Development Core Team 2016). Because the Koli and Konnevesi populations were found to be in the same cycle phase (Fig. 1), and in preliminary analyses they behaved similarly, these populations were combined in the subsequent analyses. Statistical tests for the voles from the original population were done for subsamples of voles that were randomly chosen for the experiments from the captured animals in their original sites. However, at low densities due to low numbers of captured voles in their original site all available voles were used in the experiments and taken into the analyses.

Original head widths and weights at capture were analyzed with analysis of variance (ANOVA); Origin [two levels; North (= Muhos) and South (= Koli and Konnevesi)], Sex and their interaction were used in the full model.

All other analyses were conducted by mixed effects models with library nlme (Pinheiro et al. 2010) and, in the case of data with a binary distribution, lme4 (Bates et al. 2012). Model simplification was done by choosing and reporting the best model from the set of predefined models based on the anova command in R. The maximum likelihood method was used for comparing models and restricted maximum likelihood (REML) in the final model to obtain the model estimates. Full model contained Sex in the interaction with Origin. To account for pseudoreplication, enclosure was used as a random factor in all analysis after the release of the voles into the enclosures (Zuur et al. 2009).

From the head width analysis in the first experiment, one South (Koli) male was excluded as an outlier, because its’ head had increased approximately 2 mm more than the average during winter. It was interpreted as measurement or typing error.

Survival and maturation were analyzed from binary values; survived until the end of experiment or not, or mature or not, with the lme4 package with a binomial error distribution. *P* values for the factors in the best model were calculated with a likelihood ratio test using drop1 command. Significant results are marked in the result section with following symbols: non-significant result (NS), *P = 0.01–0.05, **P = 0.001–0.01 and ***P < 0.001.

Vole density estimates were analyzed from trapping data with a robust design model in R (R Development Core Team 2016) with package Rcapture (Baillargeon and Rivest 2007). A Chao m0 model was used to get the density estimates for each trapping session and for each enclosure. In addition, survival probability estimates and number of recruit estimates between trapping sessions were obtained. We did not include the first two trapping sessions, because populations had not yet started to increase. When analyzing survival, trapping session within each enclosure was used as random factor to account for the temporal pseudoreplication (Crawley 2007) and time (trapping session) was added as fixed variable. We analyzed both sexes pooled, i.e., total number of voles estimated to be alive in the enclosure.

To check the assumptions of the models, the normality of residuals and homogeneity of variance of the best models were plotted in R. The possible autocorrelation of the residuals was checked with ACF plots in R. Multicollinearity was studied with a correlation matrix and with VIF. VIF value of 5 was used as a cutoff and there was no indication of multicollinearity between covariates or autocorrelation in the residuals.

## Results

### Original wild populations

**Fig. 3:**
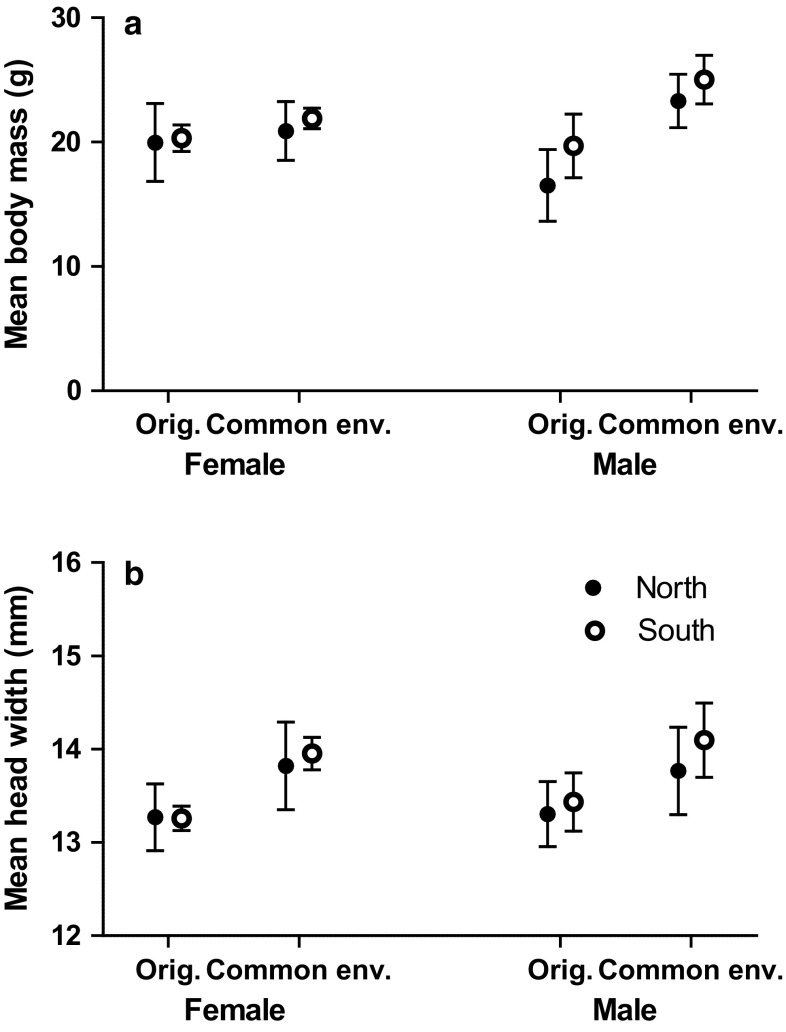
**a** The mean body mass (± 95% Cl) and **b** the mean head width (± 95% Cl) of both sexes of voles at capture (original) and when removed from enclosures in April (common environment) in the first experiment. Note that in this first experiment all individuals measured in spring were also measured in September

The dynamics of the original populations and average weights of all captured voles before, during and after the experimental periods are shown in Fig. 2. Voles for the first experiment were captured in autumn 2008 when bank voles of the North population were in the low phase and those from South in the peak phase (Fig. 2). According to expectations, voles from peak populations were 2.1 g (**) heavier than voles from low phase populations (Fig. 3; see all the statistics for original populations from ESM Tables 1 and 2). Females were 1.6 g (*) heavier than males. The interaction between Origin and Sex was also statistically significant. When tested both sexes separately, there was no difference in the body mass of the females (N 32 from South and 15 from North) between origin but males from North low population were 3.2 g (*N* = 32*) lighter than South peak population males (*N* = 17, Fig. 3). There were no statistical differences in head width in different populations (Fig. 3). Males tended to have had 0.13 mm wider heads than females but difference was not significant. There was no Origin and Sex interaction in the original head width either (Fig. 3).

**Fig. 4:**
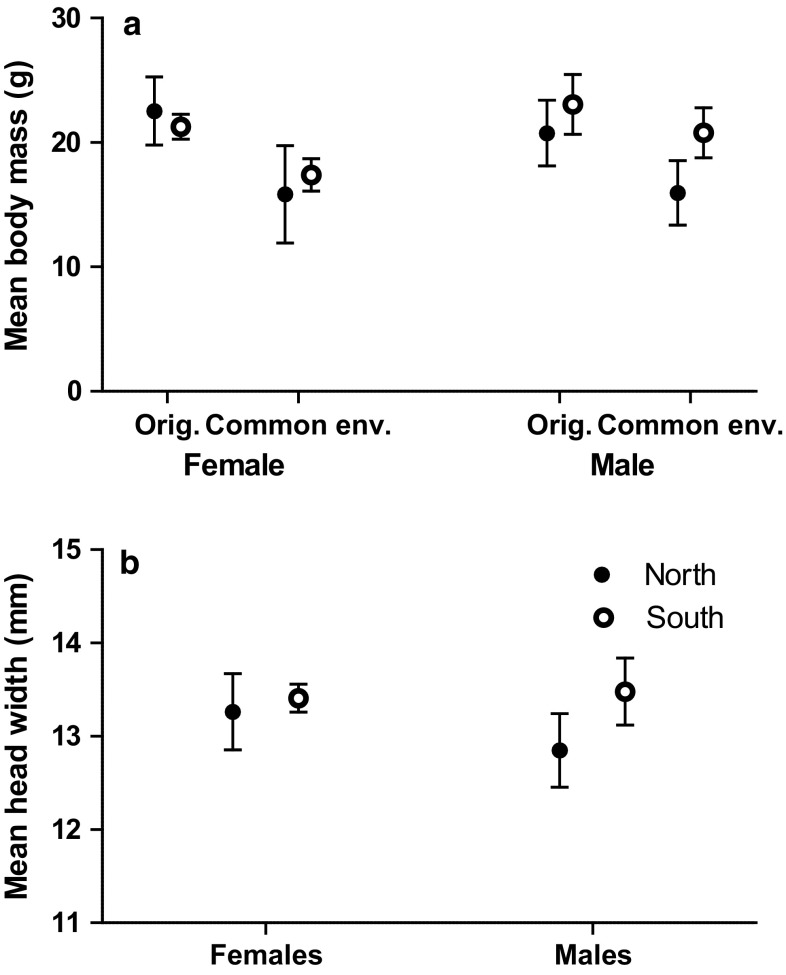
**a** The mean body mass (± 95% Cl) of both sexes of voles at capture (original) and when removed from enclosures in April (common environment) in the second experiment. Note that in this second experiment all individuals measured in spring were the first generation offspring of original individuals released in June. **b** The mean head width (± 95% Cl) of original individuals at capture

Voles for the second experiment were captured in spring 2009 when the North population had low density but it was increasing, and the South populations were declining but the densities were still higher than in the North (Fig. 2). There was a statistically significant interaction in the weight of the voles between different Origin and Sex (**, Fig. 4, South females *N* = 32, North females *N* = 16, South males *N* = 33, North males *N* = 19). This interaction was the result of 1.3 g heavier Northern increase phase females compared to Southern decline phase females (NS), while males in the North increase population were 2.3 g lighter than males in the Southern declining populations (***). We found differences in the head width of the voles with different Origins, Northern increase phase voles had 0.4 mm narrower heads than Southern decline phase voles (***, Fig. 4 South females *N* = 32, North females *N* = 17, South males *N* = 33, North males *N* = 19). There was no difference between the sexes (NS) but the interaction Origin and Sex was statistically significant (**, Fig. 4) meaning that Northern males’ heads were 0.4 mm narrower than in Northern females, while this was opposite in the South where males had 0.07 mm wider heads than females. Head width differences were not statistically significant in the females between Northern and Southern populations, but Northern increase phase males had 0.6 mm narrower heads than Southern decline phase males (***, Fig. 4).

### Overwintering experiment in the common environment: body size and overwintering success of parental populations

In this first experiment, we did not capture any weaned young but some lactating females were found in April.

Differences in vole weights remained similar over winter; voles from the North low phase were on average 1.3 g lighter than Southern peak phase voles (*, Table 1; Fig. 3). In contrast to the sex differences seen in September at capture, the males were clearly 2.8 g heavier in April than females (***, Table 1; Fig. 3).

**Table 1:** Mean ± SE of the body mass and head width of the vole individuals in April in the first experiment

Original individuals	North	*N*	South	*N*	Variable	*F*	*df*	*P*
Body mass (g)								
Females	20.9 ± 2.4	7	21.9 ± 0.8	17	Origin	1.5	1, 10	0.021
Males	23.3 ± 2.2	11	25.0 ± 2.0	21	Sex	41.1	1, 42	< 0.001
Head width (mm)								
Females	13.8 ± 0.1	11	14.00 ± 0.2	20	Origin	4.0	1, 10	0.072
Males	13.8 ± 0.4	11	14.1 ± 0.4	20				

Test statistics are presented from the best-fit model

There was a trend that Southern peak phase voles had wider heads after winter than the Northern low phase ones (Table 1; Fig. 3). No difference was observed in the overwinter survival of voles (survival of South females 62.5 ± SE 8.6%, North females 73.3 ± SE 11.8%, South males 68.8 ± SE 8.3% and North males 64.7 ± SE 11.9%).

### Breeding and overwintering in the common environment: body size, population dynamics and maturation of F1 generation

Only the founder voles of experimental populations reproduced during the summer, meaning that all the young enclosure-born individuals belonged to the F1 generation. Only one out of 96 original voles survived the winter until next April.

The weight development of the original individuals was investigated until September during which a sufficient number of them were still alive. Northern increase phase voles were 2.1 g lighter than Southern decline phase voles (*) and females were 3.6 g heavier than males (***, Table 2a).

**Table 2:** Mean ± SE of the body mass and maturation of the vole individuals in the second long term experiment

Body mass (g)	North	*N*	South	*N*	Variable	*F*	*df*	*P*
(a) Body mass of the founder individuals was measured in September when a substantial proportion of those were still alive, and mature F1 individuals after wintering in April								
Original individuals								
Females	24.9 ± 0.8	5	26.4 ± 0.5	15	Origin	6.9	1, 10	0.027
Males	20.4 ± 1.3	4	23.1 ± 0.6	11	Sex	24.9	1, 24	< 0.001
F1 individuals								
Females	15.9 ± 0.4	23	17.3 ± 0.3	53	Origin	3.1	1, 10	0.109
Males	17.8 ± 0.5	25	20.7 ± 0.3	49	Sex	96.7	1, 138	< 0.001
					Origin × sex	4.9	1, 138	0.029
F1 individuals, sexes separately								
Females	15.9 ± 0.4	23	17.3 ± 0.3	53	Origin	3.5	1, 9	0.094
Males	17.8 ± 0.5	25	20.7 ± 0.3	49	Origin	5.1	1, 10	0.047
Mature F1 individuals								
Females	19.3 ± 0.1	2	18.1 ± 0.4	23	Origin	1.4	1, 9	0.266
Males	18.7 ± 0.4	7	21.1 ± 0.3	41	Sex	43.5	1, 60	< 0.001
					Origin × sex	7.5	1, 60	0.008

Proportion of mature individuals (%)	North	*N*	South	*N*	Variable	*χ* ^2^	*df*	*P*
(b) Proportion of mature individuals in April								
Maturation of F1								
Females	8.7 ± 6.0	23	43.4 ± 6.8	53	Origin	7.8	1	0.005
Males	28.0 ± 9.2	25	85.4 ± 5.3	49	Sex	24.2	1	< 0.001

Body mass (g)	Females	*N*	Males	*N*	Variable	*F*	*df*	*P*
(c) Body mass of immature individuals in April								
Immature F1 individuals	16.4 ± 0.3	51	17.8 ± 0.5 g	26	Sex	24.9	1, 24	< 0.001

Test statistics are presented from the best-fit model

There was a statistically significant interaction in the weights of the F1 voles of different Origin and Sex in April (*, Table 2a; Fig. 4). This interaction was a result of the female offspring of Southern decline phase voles being almost the same size as the male offspring of Northern increase phase voles, while Northern F1 females were much smaller than Southern F1 females. When testing females alone, female offspring of Northern increase phase voles were 1.4 g smaller than the F1 females of Southern decline population (NS, Table 2a; Fig. 4). Northern F1 males were 2.9 g lighter compared to Southern F1 males (*, Table 2a; Fig. 4). Besides that, there were 39.5 percentage points more immature individuals in the North increase phase than in the South decline phase populations and 20 percentage points more mature males than females in both populations (Table 2b). When analyzing immature and mature individuals separately, there were no differences in weights between immature individuals from different populations. The only statistically significant difference in immature individuals was between sexes; males were 1.6 g larger than females (***, Table 2c). In mature voles, there was an interaction between Sex and Origin (**, Table 2a). This interaction was a result of Northern F1 females being 1.25 g heavier than Southern F1 females; however, there were only two mature females in the Northern population, while Northern mature males were still 2.4 g smaller than mature Southern males (Table 2a).

**Fig. 5:**
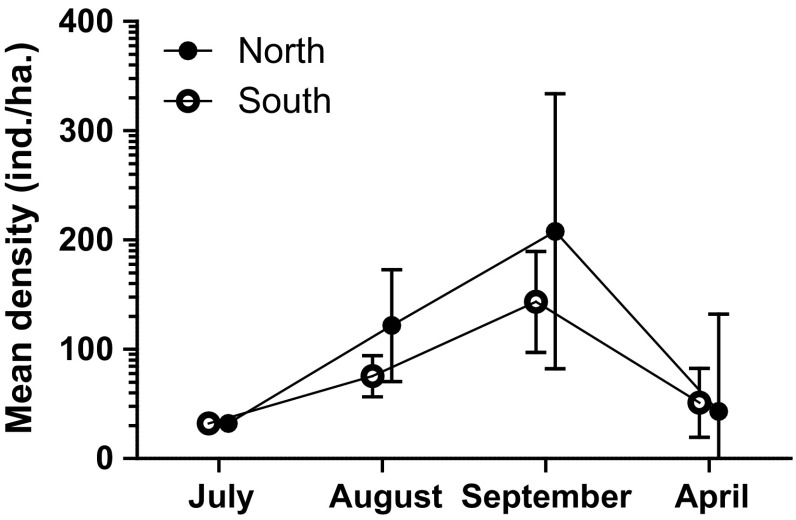
Dynamics of populations of different origin in enclosures in the first experiment. Density in 0.25 ha enclosures is converted to no. of voles/ha (± 95% Cl)

The Northern population, in which founder individuals came from increase phase, increased in size most rapidly (Time **, Origin *, Table 3a; Fig. 5). The growth of the Northern population during summer was faster than in the Southern population originating from decline phase when measured as August density (**, Table 3b; Fig. 5). In spring, there were no differences between populations in size (Fig. 5). The number of pups produced per breeding female changed over time (**) and was the highest in the Northern population (Table 3c). The number of pups born (recruits) was also higher in the Northern population than in Southern population (Origin *, Time ***, Origin and Time **, Table 3d). There was an interaction between Time and Origin in vole survival to the next trapping session (*, Time ***, Table 3e). The interaction was a result of the higher survival probability in the Northern population from July to August compared to the Southern population (NS, Table 3e; Fig. 5).

**Table 3:** Population parameters of the populations in the second long term experiment based on trapping data with robust design model analysis

Population size	Variable	*F*	*df*	*P*
(a) Population size in the enclosures				
	Origin	5.8	1, 10	0.037
	Time	30.4	1, 11	0.002

August density	Variable	*F*	*df*	*P*
(b) Population size in August				
	Origin	10.1	1, 10	0.009

*N* pups/breeding female	August	*N* females	September	*N* females	Variable	*F*	*df*	*P*
(c) Number of pups/breeding females^a^								
North	6.0 ± 0.7	31	8.9 ± 1.2	16	Origin	3.0	1, 10	0.112
South	4.3 ± 0.5	50	7.0 ± 1.0	26	Time	13.0	1, 11	0.004

Number of new pups	July to August	*N*	August to September	*N*	Variable	*F*	*df*	*P*
(d) Number of new pups in each enclosure^b^								
North	24.1 ± 2.8	96.2	33.4 ± 5.2	133.4	Origin	8.4	1, 10	0.016
South	13.9 ± 2.3	114.4	21.0 ± 3.3	167.7	Time	51.1	2, 20	< 0.001
					Time × origin	2.7	2, 20	0.089

Survival (%)	July to August	*N*	August to September	*N*	Variable	*F*	*df*	*P*
(e) Survival of voles based on robust design model estimates conducted separately for each enclosure^c^								
North	88.9 ± SE 7.6	4	62.9 ± SE 8.3	4	Origin	0.1	1, 10	0.780
South	68.9 ± SE 6.6	8	76.4 ± SE 6.5	8	Time	11.1	2, 20	< 0.001
					Time × origin	3.7	2, 20	0.043
Survival July–August					Origin	3.4	1, 10	0.096

Test statistics are based on the best-fit model with mean ± SE

^a^Sample size is the number of breeding females

^b^*N* is based on robust design model analysis

^c^*N* is the number of enclosure populations

## Discussion

Voles captured from different areas in different phases of the population density cycle had dissimilar body sizes as expected (e.g., Chitty 1967; Norrdahl and Korpimäki 2002a; Johannesen and Andreassen 2008; Sundell and Norrdahl 2002). This phase dependence in body size was clear in body mass; peak phase voles were heavier than individuals from the low phase. There was no detectable difference in head width, the second measure of individual size in additional to body mass, in autumn 2008. Next spring 2009, there was no difference in the body mass between the rising Northern population and declining Southern population females. Surprisingly, Northern males were still lighter than Southern males. The Northern population also had individuals of the narrowest heads.

### One winter is not enough to change original body size

In the first overwintering experiment, the founder individuals were measured again in spring at the end of the experiment and the voles maintained their size differences. However, although all voles had grown bigger in size, the animals originating from the peak populations (South) were still the largest despite the common overwintering conditions. There was also a similar, but non-significant, trend in the head widths. Thus, our experiment with persisting body sizes resembling the original body weight difference suggests that body size is not determined by the current non-breeding season environmental conditions. Environmental factors that were common for all populations in our experiment were, at least, food availability and quality, weather conditions, and avian predation pressure. Thus, we suggest that the body size of voles is predominantly genetically determined, or is determined already early in development (Lindström 1999). Which environmental conditions affect vole body size early in life cannot be answered with this experiment.

### The body size traits persist in breeding population and F1-generation males

The second experiment provided similar, though not always statistically significant, results regarding body size. As in the first experiment, original parent voles kept the size difference until the end of the summer, suggesting that the common breeding season environment is not sufficient to even out the size differences.

Besides that, the body size difference was transferred from parents to the F1 generation, which was clearly seen, especially in mature F1 males. The general result showing smaller size in the population from the North may be partly explained by the differences in maturation rate between populations. An interesting general life history result from the second experiment was that the F1 voles of the Northern population were still mostly immature and, therefore, small in size, in April while the voles from the South were mostly mature and, therefore, larger in size. However, when we compared mature and immature individuals separately, no difference in sizes of immature voles between populations was detected.

A marked result was that in mature males, the population difference in size remained in the F1 generation. This result again suggests, as in the first experiment, that current common conditions in our enclosures did not shape the size of individuals of different origins. Furthermore, the result suggests that early life conditions, common among enclosures, do not determine male bank voles’ body size. However, maternal effects shaping offspring size cannot be completely ruled out. Even if mothers were living in a common environment during pregnancy and lactation, they had experienced different conditions in their past lives in nature. This can possibly have had carry-over effects to their offspring size when matured (Livnat et al. 2005; Plaistow et al. 2006; Burton and Metcalfe 2014).

Why were the observed effects of the density cycle phase clearer in males? We do not have a clear answer for this question but one option could be competition for mates. Although the sex ratio of bank voles does not vary considerably during the cycle (Norrdahl and Korpimäki 2002b), it does not mean that the operational sex ratio would stay constant.

It is possible that Northern voles are on average smaller than South voles, irrespective of the cycle phase. We could not test this comprehensively as we did not have enough data on animals in comparable cycle phases. There is an indication that Northern voles are generally smaller that Southern voles, or that their size is “ceilinged” within a smaller window. However, also the density of Northern voles was lower than Southern voles in phases where we have data for comparison of size, and if size is directly dependent on density, not phase itself, it makes the comparison even more difficult. More research and data are needed to answer this question reliably.

### Are northern voles smaller only because they mature later?

The variation in maturation rate of enclosure-born individuals in spring, monitored in the second experiment, deserves itself attention. It suggests that there might be a population-level adaptation to local original conditions in bank voles. The offspring of the smaller individuals from the North had a lower maturation rate in spring than larger individuals whose parents came from more southern locations. It is likely that in the north spring comes later and thus the optimal breeding season starts later. The start of breeding may be partly genetically determined so that individuals of northern origin will start to reproduction later to be sure of favorable environmental conditions in spring (Sipari et al. 2014). Plasticity is involved in maturation as in previous studies it has been observed that the start of reproduction is affected by the density (e.g., cycle phase), nutrition, predation risk and ambient temperature (Mihok and Boonstra 1992; Prévot-Julliard et al. 1999; Eccard and Ylönen 2001; Haapakoski et al. 2012; Jochym and Halle 2012; Oksanen et al. 2012; Sipari et al. 2014). The start of breeding differs between phases of the population cycle so that during the increase phase, voles start reproduction earlier than in the peak phase (Norrdahl and Korpimäki 2002b). Our Northern population was in the increase phase, but being born and overwintering in the common environment and did not change the timing of maturation. Thus, our study verifies an overriding role of adaptation to local original conditions, which remains in a new environment until the next generation at least.

### Increase phase population grew faster also in common conditions

The most intriguing result of the breeding season experiment was the rapid population growth of the North population from the start of the experiment in June until autumn. This means that the original growth-determined or programmed population kept its potential for rapid growth compared to declining populations in common conditions. This is a similar result as in Mihok and Boonstra’s (1992) well-controlled experiment with meadow voles (*Microtus pennsylvanicus*). More rapid growth during the summer was not, however, coupled with better survival over winter and the Northern population declined by next spring to same level as the other populations.

As none of individuals in these populations reproduced during winter, the decline was solely due to mortality, which was more intense in the Northern population. One possible reason for the rapid decline of the Northern population was the lowered carrying capacity of enclosures in winter due to (over)grazing by the dense previous summer population. It is also possible that the grazing during the first experiment, which ended in April, more than 1 month before this second experiment, has affected the population growth, especially the fast population growth of Northern voles. However, the densities in the first experiment were relatively low and similar between enclosures, which makes this possibility unlikely. This conclusion is further supported by the experiment of Klemola et al. (2000) in which previous heavy grazing by voles did not affect negatively the population growth of subsequent populations. Another explanation is that conditions for Northern voles in the Konnevesi region would have been better for them than for Southern voles, which originated in the area (Konnevesi) or nearby (Koli), and this would have been facilitated better growth of the Northern population. This explanation cannot be ruled out, but usually animals do better in the regional conditions they are adapted to than elsewhere, if natural enemies are the same, like in this case where the maximum distance between sites was 260 km without any major isolation barriers.

The rapid population growth during the breeding season could be explained by heredity, maternal or early life effects. Only the original population voles reproduced during the summer. Thus, the increase was solely the result of the effective reproduction of founder Northern voles that had experienced the conditions of an increasing population in their early life. There was a tendency for larger litters in the North, measured as number of recruits per female, and clear differences in total number of recruits. In addition, survival was better in the Northern population compared to others during summer. However, this turned to common low survival over the subsequent winter.

## Conclusion

Ergon et al. (2001b) found that field voles (*M. agrestis*) moved to common laboratory conditions kept their reproductive traits (frequency of reproduction, proportion breeders and litter size) observed in the field. The same was true in Mihok and Boonstra’s (1992) experiment with meadow voles. Both studies also emphasized the importance of early life effects on their observation. However, Ergon et al.’s result showed that reproductive differences observed between peak and increase populations vanished in the next generation. The first result of this study is in line with ours, as we noticed that voles from an increasing population kept the high reproductive potential in common environmental conditions. On the other hand, we observed later maturation of F1 originating from an increasing population. This strongly suggests that the timing of maturation was not changed in one generation in the common environment.

In another experiment by Ergon et al. (2001a) transplanted field voles resembled the local population in body size, and maturation, after one overwintering. This is in contradiction to our results from the first overwintering experiment. Our voles’ original body size difference remained over winter, in the parental breeding population of the next experiment, and also in mature F1 voles. The main differences between Ergon et al. (2001a, b) and our experiments were that we used confined enclosures without marked density effects and lack of mammalian predators. These two key aspects, competition and predation, in the immediate biotic environments of voles can be the key factors affecting vole’s body size in cyclic populations in nature.

Based on our experiments, we can exclude the effects of current climatic conditions, food availability and predation by avian predators as mechanisms causing the evident phase-dependence in body size, reproduction (i.e., reproductive potential) and survival. We were able to verify the results in two independent experiments conducted with autumn-captured or spring-captured founder voles. Our study emphasizes the importance of the early life environment and maternal effects, as well as possible genetic component in the life history traits of cyclic bank voles. More controlled experiments are needed in the future to investigate the relative roles of these mechanisms and to find out the most important past environmental factors affecting currently observed life history traits.

## Electronic supplementary material

Below is the link to the electronic supplementary material.

Supplementary material 1 (DOCX 20 kb)

## References

[CR1] Baillargeon S, Rivest LP (2007). Rcapture: loglinear models for capture-recapture in R. J Stat Softw.

[CR2] Bates D, Maechler M, Bolker B (2012) lme4: linear mixed-effects models using S4 classes. http://cran.rproject.org/web/packages/lme4/index.html

[CR3] Boonstra R, Krebs CJ (1979). Viability of large and small-sized adults in fluctuating vole populations. Ecology.

[CR4] Burton T, Metcalfe NB (2014). Can environmental conditions experienced in early life influence future generations?. Proc R Soc B.

[CR5] Chitty D (1952). Mortality among voles (*Microtus agrestis*) at Lake Vyrnwy, Montgomeryshire in 1936–9. Philos Trans R Soc B.

[CR6] Chitty D (1967). The natural selection of self-regulatory behaviour in animal populations. Proc Ecol Soc Aust.

[CR7] Crawley MJ (2007). The R book.

[CR8] DeWitt TJ, Scheiner SM (2004). Phenotypic plasticity.

[CR9] Eccard JA, Ylönen H (2001). Initiation of breeding after winter in bank voles: effects of food and population density. Can J Zool.

[CR10] Ergon T, Lambin X, Stenseth NC (2001). Life-history traits of voles in a fluctuating population respond to the immediate environment. Nature.

[CR11] Ergon T, MacKinnon JL, Stenseth NC, Boonstra R, Lambin X (2001). Mechanisms for delayed density-dependent reproductive traits in field voles, *Microtus agrestis*: the importance of inherited environmental effects. Oikos.

[CR12] Haapakoski M, Sundell J, Ylönen H (2012). Predation risk and food: opposite effects on overwintering survival and onset of breeding in a boreal rodent. J Anim Ecol.

[CR13] Hanski I, Henttonen H, Korpimäki E, Oksanen L, Turchin P (2001). Small-rodent dynamics and predation. Ecology.

[CR14] Hansson L (1983). Competition between rodents in successional taiga forest: *Microtus agrestis* vs. *Clethrionomys glareolus*. Oikos.

[CR16] Hansson L, Henttonen H (1988). Rodent dynamics as community processes. Trends Ecol Evol.

[CR17] Hansson L, Jaarola M (1989). Body size related to cyclicity in microtines: dominance behaviour or digestive efficiency?. Oikos.

[CR18] Helle H, Koskela E, Mappes T (2012). Life in varying environments: experimental evidence for delayed effects of juvenile environment on adult life history. J Anim Ecol.

[CR19] Hellstedt P, Sundell J, Helle P, Henttonen H (2006). Large-scale spatial and temporal patterns in population dynamics of the stoat *Mustela erminea* and the least weasel *M. nivalis* in Finland. Oikos.

[CR20] Huitu O, Koivula M, Korpimäki E, Klemola T, Norrdahl K (2003). Winter food supply limits growth of northern vole populations in the absence of predation. Ecology.

[CR21] Jochym M, Halle S (2012). To breed or not to breed? Predation risk induces breeding suppression in common voles. Oecologia.

[CR22] Johannesen E, Andreassen HP (2008). Density-dependent variation in body mass of voles. Acta Theriol.

[CR23] Johnsen K, Boonstra R, Boutin S, Devineau O, Krebs CJ, Andreassen HP (2017). Surviving winter: food, but not habitat structure, prevents crashes in cyclic vole populations. Ecol Evol.

[CR24] Klemola T, Koivula M, Korpimäki E, Norrdahl K (2000). Experimental tests of predation and food hypotheses for population cycles of voles. Proc R Soc B.

[CR25] Korpimäki E, Norrdahl K, Huitu O, Klemola T (2005). Predator-induced synchrony in population oscillations of coexisting small mammal species. Proc R Soc B.

[CR26] Krebs CJ, Myers J (1974). Population cycles in small mammals. Adv Ecol Res.

[CR27] Lidicker WZ, Ostfeld RS (1991). Extra-large body size in California voles: causes and fitness consequences. Oikos.

[CR28] Lindström J (1999). Early development and fitness in birds and mammals. Trends Ecol Evol.

[CR29] Livnat AS, Pacala W, Levin SA (2005). The evolution of intergenerational discounting in offspring quality. Am Nat.

[CR30] Mihok S, Boonstra R (1992). Breeding performance in captivity of meadow voles (*Microtus pennsylvanicus*) from decline-and increase-phase populations. Can J Zool.

[CR31] Monaghan P (2008). Early growth conditions phenotypic development and environmental change. Philos Trans R Soc B.

[CR32] Norrdahl K, Korpimäki E (2002). Changes in individual quality during a 3-year population cycle of voles. Oecologia.

[CR33] Norrdahl K, Korpimäki E (2002). Changes in population structure and reproduction during a 3-yr population cycle of voles. Oikos.

[CR34] Oksanen TA, Koivula M, Koskela E, Mappes T, Soulsbury CD (2012). Interactive effects of past and present environments on overwintering success—a reciprocal transplant experiment. Ecol Evol.

[CR35] Oli MK (1999). The Chitty effect: a consequence of dynamic energy allocation in a fluctuating environment. Theor Popul Biol.

[CR36] Pinheiro J, Bates D, DebRoy S, Sarkar D (2010) The R Development Core Team nlme: linear and nonlinear mixed effects models. R package version 31-97

[CR37] Plaistow SJ, Lapsley CT, Benton TG (2006). Context-dependent intergenerational effects: the interaction between past and present environments and its effect on population dynamics. Am Nat.

[CR38] Prévot-Julliard A-C, Henttonen H, Yoccoz NG, Stenseth NC (1999). Delayed maturation in female bank voles: optimal decision or social constraint. J Anim Ecol.

[CR39] R Development Core Team (2016) R: A language and environment for statistical computing. R Foundation for Statistical Computing. Vienna. Austria. ISBN 3-900051-07-0. https://www.R-projectorg/. Accessed 11 Oct 2018

[CR40] Schlichting CD (1986). The evolution of phenotypic plasticity in plants. Annu Rev Ecol Syst.

[CR41] Sipari S, Haapakoski M, Klemme I, Sundell J, Ylönen H (2014). Sex-specific variation in the onset of reproduction and reproductive trade-offs in a boreal small mammal. Ecology.

[CR42] Sundell J, Norrdahl K (2002). Body size-dependent refuge in voles: an alternative explanation of the Chitty effect. Ann Zool Fenn.

[CR43] Sundell J, Huitu O, Henttonen H, Kaikusalo A, Korpimäki E, Pietiäinen H, Saurola P, Hanski I (2004). Large-scale spatial dynamics of vole populations in Finland revealed by the breeding success of vole-eating avian predators. J Anim Ecol.

[CR44] Sundell J, Church C, Ovaskainen O (2012). Spatio-temporal patterns of habitat use in voles and shrews modified by density season and predators. J Anim Ecol.

[CR45] Sundell J, O’Hara RB, Helle P, Hellstedt P, Henttonen H, Pietiäinen H (2013). Numerical response of small mustelids to vole abundance: delayed or not?. Oikos.

[CR46] Taborsky B (2006). Mothers determine offspring size in response to own juvenile growth conditions. Biol Lett.

[CR47] Whitman DW, Ananthakrishnan TN (2009). Phenotypic plasticity of insects: mechanisms and consequences.

[CR48] Yoccoz NG, Mesnager S (1998). Are alpine bank voles larger and more sexually dimorphic because adults survive better?. Oikos.

[CR49] Zuur AF, Ieno EN, Walker NJ, Saveliev AA, Smith GM (2009). Mixed effects models and extensions in ecology with R.

