# Supplementary material for: Do phase-dependent life history traits in cyclic voles persist in a common environment?
Source: Oecologia. 2019 May 7;190(2):399–410. doi: 10.1007/s00442-019-04410-3 (PMC6571100; doi:10.1007/s00442-019-04410-3)
Supplement: Supplementary file 1 — Supplementary material 1 (DOCX 20 kb) [file 442_2019_4410_MOESM1_ESM.docx]

ESM Sundell et al. Oecologia

Body weight and head width of the original individuals used in the experiment.

# Experiment 1

Mean ± SE of the body mass and head width of the original vole individuals before experiment. Table 1a) original weight of both sexes. Table 1b and c) weight of females and males separately. Table 1d) head width of original voles. Test statistic are presented from the analysis of variance.

| Weight |  | Variable | F | df | P |
| --- | --- | --- | --- | --- | --- |
|  |  | Origin | 9.24 | 1,86 | 0.003 |
|  |  | Sex | 5.27 | 1,86 | 0.024 |
|  |  | Sex * Origin | 4.15 | 1,86 | 0.045 |

Table 1a. Original weight of both sexes

| Weight females |  | Variable | T | df | P |
| --- | --- | --- | --- | --- | --- |
|  |  | Origin | 0.21 | 13,07 | 0.834 |

Table 1b. Original weight of females

| Weight males |  | Variable | T | df | P |
| --- | --- | --- | --- | --- | --- |
|  |  | Origin | 5.94 | 37,2 | < 0.001 |

Table 1c. Original weight males

| Head width |  | Variable | F | df | P |
| --- | --- | --- | --- | --- | --- |
|  |  | Origin | 0.53 | 1,92 | 0.469 |
|  |  | Sex | 2.89 | 1,92 | 0.092 |
|  |  | Sex * Origin | 0.80 | 1,92 | 0.375 |

Table 1d. Original head width of both sexes

# Experiment 2

Mean ± SE of the body mass and head width of the original vole individuals before experiment. Table 2a) original weight of both sexes. Table 2b and c) weight of females and males separately. Table 2d) head width of original voles. Table 2e and f) head width of original females and males separately. Test statistic are presented from the analysis of variance.

| Weight |  | Variable | F | df | P |
| --- | --- | --- | --- | --- | --- |
|  |  | Origin | 1.08 | 1,96 | 0.301 |
|  |  | Sex | 0.97 | 1,96 | 0.327 |
|  |  | Sex * Origin | 8.94 | 1,96 | 0.004 |

Table 2a. Original weight of both sexes

| Weight females |  | Variable | T | df | P |
| --- | --- | --- | --- | --- | --- |
|  |  | Origin | -1.25 | 33.03 | 0.219 |

Table 2b. Original weight of females

| Weight males |  | Variable | T | df | P |
| --- | --- | --- | --- | --- | --- |
|  |  | Origin | 3.95 | 47.42 | < 0.001 |

Table 2c. Original weight of males

| Head width | Variable | F | df | P |
| --- | --- | --- | --- | --- |
|  | Origin | 20.78 | 1,96 | < 0.001 |
|  | Sex | 1.36 | 1,96 | 0.247 |
|  | Sex * Origin | 7.36 | 1,96 | 0.008 |

Table 2d. Original head width both sexes

| Head width females | Variable | T | df | P |
| --- | --- | --- | --- | --- |
|  | Origin | 0.78 | 20.08 | 0.445 |

Table 2e. Original weight females

| Head width males | Variable | T | df | P |
| --- | --- | --- | --- | --- |
|  | Origin | 6.40 | 30.16 | < 0.001 |

Table 2f. Original weight males
